# Long-term efficacy of dexamethasone treatment *via* tympanic antrum catheterization for intractable Meniere's disease

**DOI:** 10.3389/fneur.2022.1056724

**Published:** 2022-12-02

**Authors:** Yafeng Lyu, Jia Guo, Xiaofei Li, Huirong Jian, Yawei Li, Jing Wang, Zhaomin Fan, Haibo Wang, Daogong Zhang

**Affiliations:** ^1^Department of Otolaryngology-Head and Neck Surgery, Shandong Provincial ENT Hospital, Shandong University, Jinan, China; ^2^Shandong Provincial Vertigo and Dizziness Medical Center, Jinan, China; ^3^Laboratory of Vertigo Disease, Shandong Institute of Otorhinolaryngology, Jinan, China

**Keywords:** vestibular plugging, otolith dysfunction, Meniere's disease, drop attack, semicircular canal plugging

## Abstract

**Objective:**

To explore the long-term efficacy and safety of dexamethasone treatment *via* tympanic antrum catheterization (TAC) in intractable Meniere's disease (MD).

**Methods:**

In this retrospective analysis, 60 unilateral intractable MD patients treated with TAC in our hospital from January 2020 to August 2020 were followed for 2 years. Fifty patients who underwent endolymphatic sac decompression (ESD) and 50 patients who accepted intratympanic steroids (ITS) were established as the control groups. Vertigo control, hearing level, tinnitus, aural fullness and functional level were assessed during the study.

**Results:**

The effective vertigo control rate of intractable MD patients with TAC treatment was 76.7% (46/60) after 2 years follow-up, with a complete control rate of 58.3% (35/60) and a substantial control rate of 18.3% (11/60). The vertigo control rate of TAC was comparable to that of ESD (*χ*^2^ = 0.313, *p* > 0.05), and significantly higher than that of ITS (*χ*^2^ = 4.380, *p* < 0.05). The hearing loss rate of these patients was 10.8% (4/37), which was not significantly different from the control groups (*χ*^2^ = 2.452, *p* > 0.05). The tinnitus improvement rate of patients with TAC was 56.7% (34/60), which was significantly higher than that of patients with ESD (*χ*^2^ =11.962, *p* < 0.001) and ITS (*χ*^2^ =15.278, *p* < 0.001). The aural fullness improvement rate in the TAC group was 56.7% (34/60), which was significantly higher than that in the ESD (*χ*^2^ = 11.962, *p* < 0.001) and ITS groups (*χ*^2^ = 5.635, *p* < 0.05). The functional level improvement rate in the TAC group was 71.7% (43/60), which was much higher than that in the ITS group (*χ*^2^ = 17.256, *p* < 0.001), but there was no significant difference between TAC and ESD (*χ*^2^ = 0.410, *p* > 0.05). No patients had complications or adverse reactions following TAC treatment.

**Conclusion:**

Dexamethasone treatment *via* TAC can effectively control vertigo attacks and improve related symptoms of intractable MD patients, providing valuable new insights into the treatment of MD.

## Introduction

Meniere's disease (MD) is an inner ear disease with idiopathic endolymphatic hydrops, characterized by intermittent vertigo attacks, sensorineural hearing loss, tinnitus, and/or aural fullness. Its prevalence varies from 0.513 to 3.5%, and MD is most commonly seen in adults aged 40–60 years, with a female predominance (1). Most patients have unilateral onset of MD, but ~25–45% of patients progress to bilateral MD as the disease progresses (2). Devastating vertigo attacks, progressive hearing loss, and the resulting psychosomatic problems such as anxiety and depression make MD a disabling disease that seriously affects patients' daily living, social life, and work.

Since the definitive etiology and pathogenesis are still unknown, there is no cure for MD. At present, treatment is mainly based on symptomatic measures to reduce vertigo attacks preserve hearing and improve vestibular function (3). Steroid hormones, diuretics, betahistine, and other medical treatments for MD can relieve ~80% of patients with vertigo (1). Surgical procedures were considered when medical treatment failed. Endolymphatic sac decompression (ESD) is the most commonly used non-destructive procedure for intractable MD. A recent study reported the vertigo control rate among different endolymphatic sac surgery is 70–88.6%, only in favor of MD patients at early or middle stages (4). Despite the high rate of vertigo control, post-operative complications such as cerebrospinal fluid leakage, total deafness and intracranial infection may occur following vestibular neurectomy (5). As a completely destructive surgery, labyrinthectomy is only recommended for patients with severe to profound sensorineural hearing loss. As a last resort, labyrinthectomy is only recommended for patients with total deafness due to complete destruction of inner ear function (6). Additionally, the vertigo control rate of triple semicircular canal plugging reported in our previous study (7) was excellent at 98.7%, although 26.3% of the patients had hearing loss.

Intratympanic drug delivery is an alternative approach to MD (8). Intratympanic gentamicin (ITG), is known as chemical labyrinthectomy, with a vertigo control rate up 96.8% but accompanied by 0–75% hearing impairment (9, 10). Growing evidence has indicated intratympanic steroids (ITS) provide an effective alternative to ITG in symptom control, especially multi-session ITS (11, 12). However, repeated injections *via* tympanic puncture can cause burning and painful sensation (8). Moreover, the rapid loss of fluids through the Eustachian tube results in low drug absorption, unstable effects, and individual differences (13, 14). Therefore, we conceived a new type of topical medication, tympanic antrum catheterization (TAC), to treat intractable MD. Repeated intra-aural drug infusion through post-auricular placement effectively controlled vertigo attacks and showed a positive effect in preventing hearing loss and improving related symptoms.

The aim of this study was to evaluate the long-term efficacy of TAC and to compare it with ESD and ITS to determine its therapeutic value for intractable MD.

## Materials and methods

### Patients

This study retrospectively analyzed 60 patients (27 men, 34 women; aged 51.8 ± 12.4 years, with a disease duration of 50.2 ± 76.1 months) who accepted TAC treatment in the vertigo department of our hospital from January 2020 to August 2020. Fifty patients who underwent ESD (22 men, 28 women; aged 55.2 ± 11.0 years, with a disease duration of 57.8 ± 47.1 months) and 50 patients who underwent ITS (23 men, 27 women; aged 52.7 ± 13.2 years, with a disease duration of 43.1 ± 59.0 months) in the same period were allocated to the control groups. These patients were clinically diagnosed with definite MD based on the criterion of the Barany society (15). All patients continued to experience recurrent vertigo (at least 2 attacks per month, each attack lasting more than 20 min), even after at least 6 months of medical treatment (betahistine 12 mg tid and hydrochlorothiazide 25 mg bid).

Referring to previous study (16), other vestibular disorders or vertigo diseases were excluded by a series of examinations including auditory examination, vestibular function examinations and magnetic resonance imaging. All patients were followed up for 2 years. The evaluation of therapeutic effects consisted of vertigo control, hearing level, as well as the improvement of tinnitus, aural fullness, and functional level.

This study was approved by the Ethics Committee of Shandong Provincial ENT Hospital, and informed consents were obtained from all patients.

### Dexamethasone treatment *via* TAC

**Figure 1:**
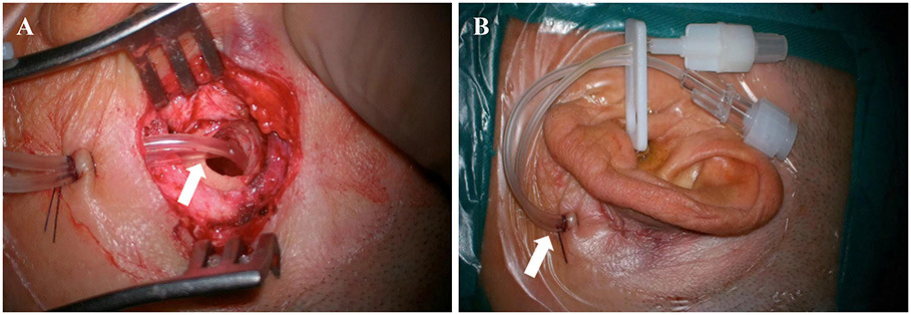
Intraoperative **(A)** and postoperative **(B)** pictures of tympanic antrum catheterization (arrows).

TAC was performed using a post-auricular approach under local anesthesia. An incision ~3 cm in length was made on the upper part of the retroauricular sulcus. After exposing the mastoid cortex, an electric drill was used to make a bone window of about 1.5–2 cm in diameter directly to the tympanic antrum. Two catheters (part of the infusion set, 3 mm in diameter) were placed in the tympanic antrum, tunneled subcutaneously and secured with 3–0 silk suture (Figure 1A). One catheter was for drug delivery and the other for regulation of the middle ear pressure during drug delivery. The incision was closed, and the external openings of the catheters were sealed and fixed in the preauricular region (Figure 1B). Dexamethasone sodium phosphate (Xinhua Pharmaceutical Co., LTD., Zibo, Shandong, China) 5 mg (1 ml) was administered through the catheter once a day for a total of 7 days. Patients were instructed to stay still in bed without talking or swallowing for at least half an hour after every injection.

### Surgical procedure of ESD

ESD was performed using a post-auricular approach under general anesthesia. An incision was made ~1 cm behind the retroauricular sulcus and was ~7 cm in length. After a standard mastoidectomy, anatomic landmarks, including the sigmoid sinus, horizontal semicircular canal, and middle cranial fossa meninges, were recognized. The endolymphatic sac was located between the sigmoid sinus and posterior semicircular canal and was usually below the plane of the horizontal semicircular canal. A grinding drill was used to remove the bone and fully expose the endolymphatic sac. Finally, the incision was closed, and the surgery was completed.

### Intratympanic steroid

Intratympanic injections were administered under surface anesthesia. About 0.4–0.6 ml dexamethasone sodium phosphate (Xinhua Pharmaceutical Co., LTD., Zibo, Shandong, China) diluted with normal saline (4:1 in volume) was injected into the tympanic cavity through the anterior inferior part of the tympanic membrane. The patients were instructed to stay still in bed without talking or swallowing for at least half an hour after each injection. Three injections during a period of 1 week (the 1st, 4th, and 7th day) were administered to each patient.

### Evaluation of vertigo

According to the 1995 criteria set by American Academy of Otolaryngology-Head and Neck Surgery (AAO-HNS) (17), a definitive spell of vertigo lasting more than 20 min was regarded as a Meniere's vertigo attack. The numerical value of the formula (the average number of vertigo attacks per month from 18 to 24 months after therapy× 100 /the average number of vertigo attacks per month for the 6 months before therapy) divided vertigo control into six scales: A (0), B (1–40), C (41–80), D (81–120), E (>120), and F (secondary treatment). Scale A (complete control) and B (substantial control) were regarded as effective vertigo control.

In addition, the dizziness handicap inventory (DHI, 0–100 scale) was used to evaluate the physical, emotional and functional effects of this disorder (18). The vertigo symptom scale (VSS; 0–60) was used to quantify the severity of vertigo (19).

### Evaluation of hearing

The four-tone average of thresholds at 0.5, 1, 2, and 3 kHz was adopted to determinate the hearing change as recommended by AAO-HNS (17). The worst hearing level of the affected ear during the previous 6 months was compared with that during the following 18–24 months. A hearing level change ≥10 dB was considered “better” or “worse” and change <10 dB was considered as “no change.”

### Evaluation of tinnitus

Similar to DHI, the tinnitus handicap inventory (THI, 0–100 scale) (20) was used for self-assessment of tinnitus disorder. The change in THI score ≥20 was considered as “better” or “worse” and change <20 was considered as “no change.”

### Evaluation of aural fullness

Visual analog scale (VAS, 0–10 scale) was used to quantify the symptoms of aural fullness (21). The change in VAS score ≥2 was defined as “better” or “worse,” and a change of <2 was defined as “no change.”

### Evaluation of functional impairment and disability

A six-point functional level scale (FLS) was used to assess the effects of episodic vertigo on daily activities as per the 1995 AAO-HNS criteria (17). The change of FLS score ≥1 was designated as “better” or “worse” and change <1 was designated as “no change.”

### Statistics

All statistical analyses were completed using SPSS 27.0 software (SPSS Inc., Chicago, IL, USA). One-way ANOVA and two independent-sample *t*-test were used to compare the mean values among the groups. Comparisons of proportions among groups were performed applying Chi-Squared test. *P*-value (set at correction) < 0.05 was considered statistically significant.

## Results

### Clinical information

As shown in Table 1, there were no significant differences in demographic and baseline characteristics among the TAC, ESD, and ITS groups (*p* > 0.05).

**Table 1:** Demographics and baseline characteristics.

	**TAC (*n* = 60)**	**ESD (*n* = 50)**	**ITS (*n* = 50)**	**Statistic value**	***P*-value**
Age, Mean ± SD	51.8 ± 12.4	55.2 ± 11.0	52.7 ± 13.2	*F* = 1.089	>0.05
Gender				*χ*^2^ = 0.040	>0.05
Male	27 (45%)	22 (44%)	23 (46%)		
Female	33 (55%)	28 (56%)	27 (54%)		
Duration (months), Mean ± SD	50.2 ± 76.1	57.8 ± 47.1	43.1 ± 59.0	*F* = 0.687	>0.05
Pure-tone average (dB), Mean ± SD	47.2 ± 15.0	48.6 ± 14.1	47.2 ± 21.6	*F* = 0.108	>0.05
Speech discrimination (%), Mean ± SD	62.9 ± 25.2	65.8 ± 20.1	63.3 ± 28.2	*F* = 0.200	>0.05
Abnormal rate of vestibular function tests
Caloric test	49.1% (27/55)	61.4% (27/44)	39.0% (16/41)	*χ*^2^ = 4.267	>0.05
cVEMP	50.0% (29/58)	48.9% (23/47)	60.9% (28/46)	*χ*^2^ = 1.665	>0.05
oVAMP	60.3% (35/58)	76.6% (36/47)	69.2% (31/46)	*χ*^2^ = 3.129	>0.05
vHIT	35.7% (20/56)	28.3% (13/46)	25.0% (11/44)	*χ*^2^ = 1.456	>0.05

TAC, tympanic antrum catheterization; ESD, endolymphatic sac decompression; ITS, intratympanic steroid; cVEMP, cervical vestibular evoked myogenic potential; oVAMP, ocular vestibular evoked myogenic potential; vHIT, video-head impulse test.

### Vertigo control

**Figure 2:**
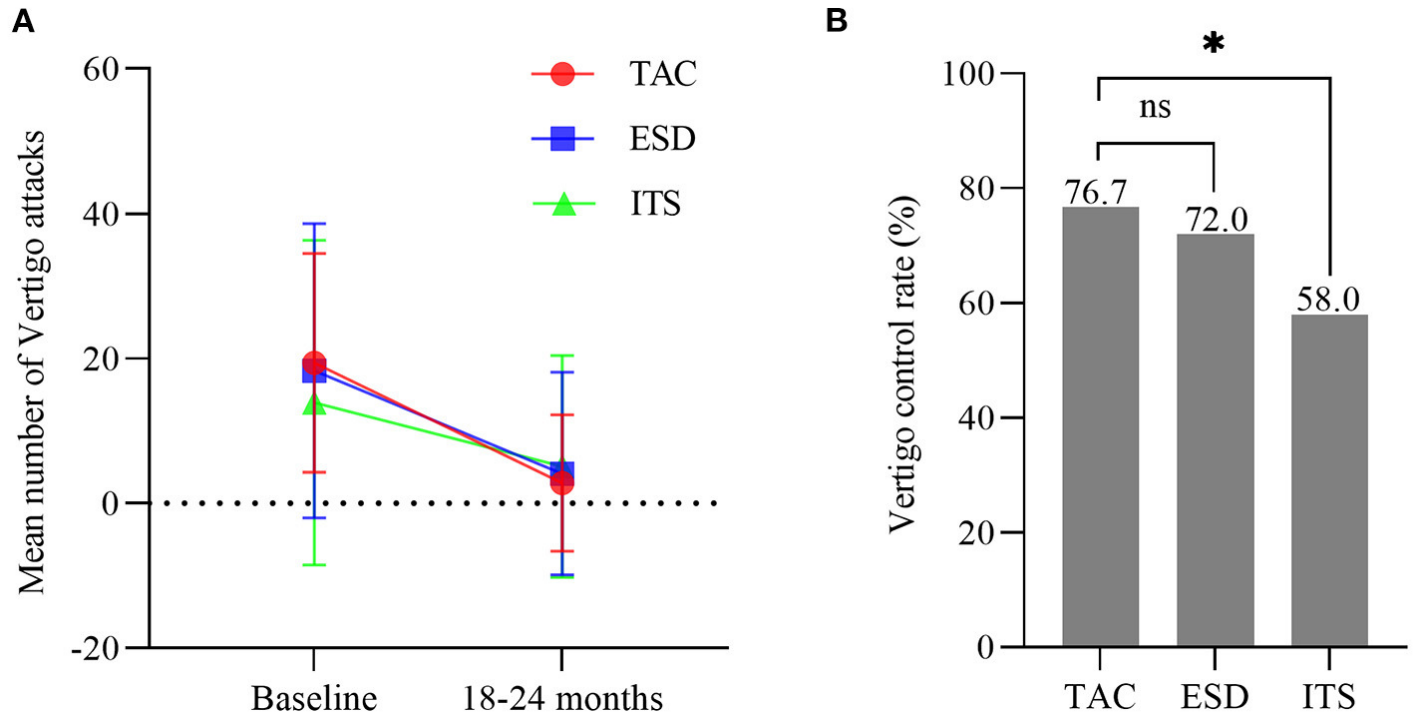
**(A)** Mean number of attacks of vertigo 6 months before treatment and in the final 6 months (bars are SDs). **(B)** The control rate of vertigo in each group (*p < 0.05). TAC, tympanic antrum catheterization; ESD, endolymphatic sac decompression; ITS, intratympanic steroid.

**Figure 3:**
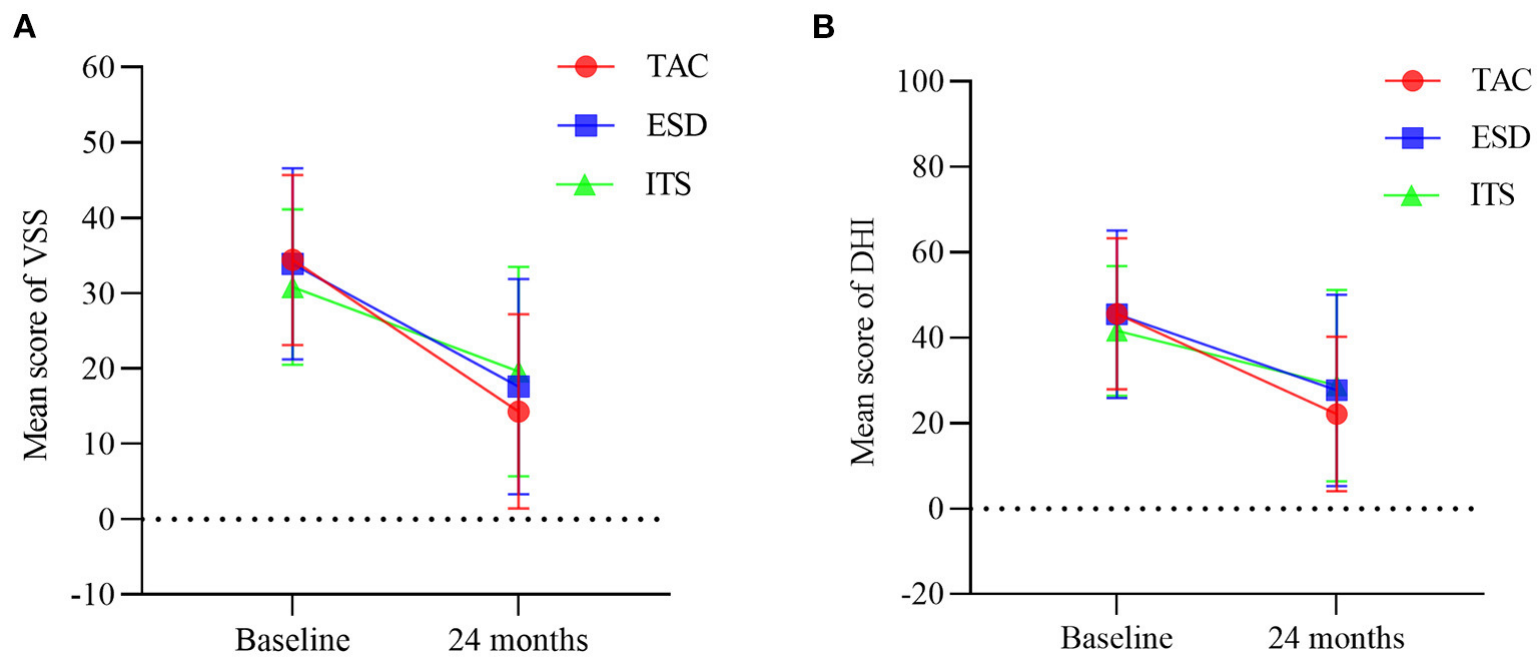
Mean scores for **(A)** vertigo symptom scale and **(B)** dizziness handicap inventory at baseline and 24 months (bars are SDs). TAC, tympanic antrum catheterization; ESD, endolymphatic sac decompression; ITS, intratympanic steroid.

Pre-operatively, there were no significant difference among the three groups regarding the frequency of vertigo attacks (*F* = 1.193, *p* > 0.05), DHI score (*F* = 0.880, *p* > 0.05), and VSS score (*F* = 1.488, *p* > 0.05). A decrease in the number of vertigo attacks was noticed in all groups 2 years after the treatment (Figure 2A). The number of vertigo attacks during 6 months in the TAC group decreased from 19.4 ± 15.1 to 2.8 ± 9.4 (*t* = 7.074, *p* < 0.001). However, no significant difference in the number of vertigo attacks in the final 6 months among the three groups was discovered (*F* = 0.361, *p* > 0.05). Notably, the effective vertigo control rate in patients with TAC was 76.7% (46/60) at the 2-year follow-up, with a complete control rate of 58.3% (35/60) and a substantial control rate of 18.3% (11/60). In the ESD and ITS groups, vertigo attacks were effectively controlled in 72.0 and 58.0% of the patients, respectively. The vertigo control rate in the TAC group was significantly higher than that in the ITS group (*χ*^2^ = 4.380, *p* < 0.05; Figure 2B), but there was no significant difference between TAC and ESD (*χ*^2^ = 0.313, *p* > 0.05; Figure 2B). Additionally, all treatments reduced the mean DHI and VSS scores during follow-up (Figure 3). For patients with TAC, mean scores decreased significantly over time for DHI (decreased from 45.6 ± 17.7 to 22.1 ± 18.1; *t* = 7.215, *p* < 0.001) and VSS (decreased from 34.4 ± 11.3 ± 17.7 to 14.3 ± 12.9; *t* = 9.106, *p* < 0.001). However, we did not observe significant differences among the three groups for DHI score (*F* = 1.674, *p* > 0.05) and VSS score (*F* = 2.167, *p* > 0.05) at 24 months.

### Hearing change

**Figure 4:**
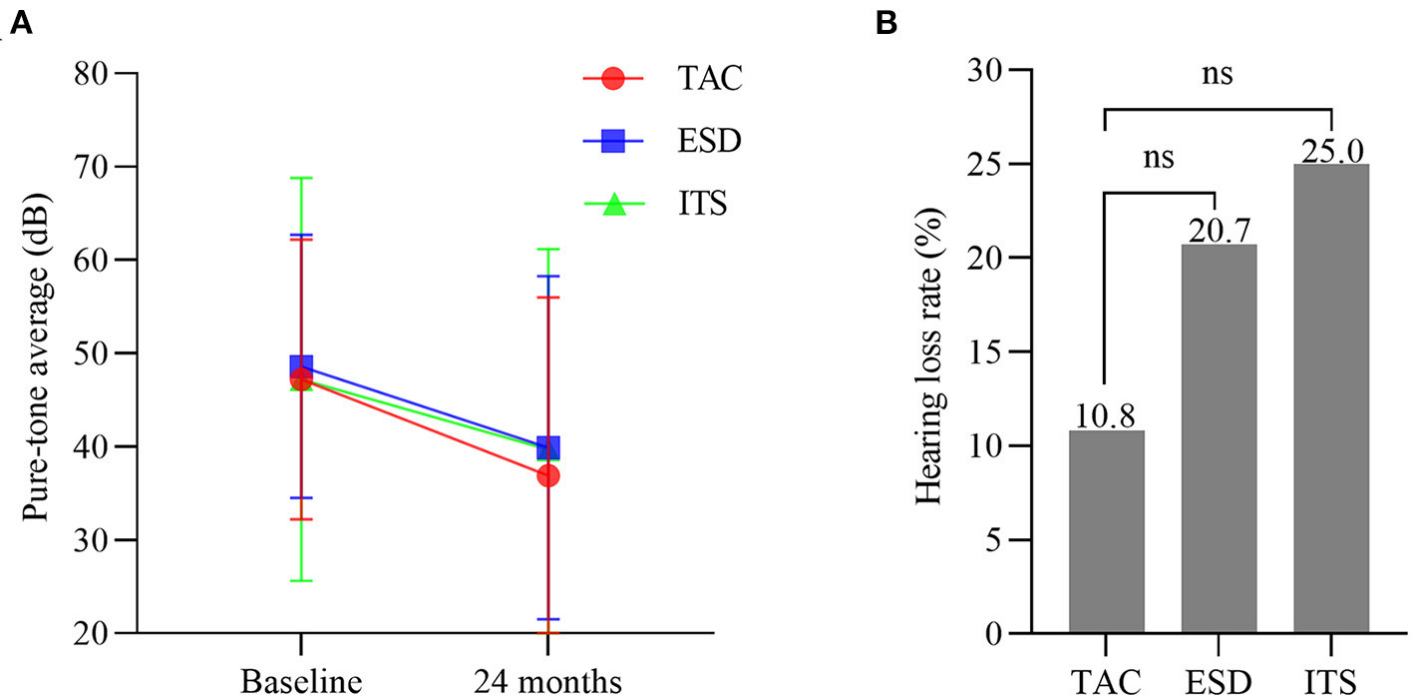
**(A)** Hearing level at baseline and 24 months (bars are SDs). **(B)** The hearing loss rate in each group. TAC, tympanic antrum catheterization; ESD, endolymphatic sac decompression; ITS, intratympanic steroid.

Over the 2 years follow-up, the pure-tone average of patients with TAC improved from 47.2 ± 15.0 to 37.1 ± 18.9 (*t* = 2.960, *p* < 0.01; Figure 4A), but without a significant difference in PTA at 24 months among the three groups (*F* = 0.255, *p* >0.05). The hearing loss rate in the TAC group was 10.8% (4/37), while hearing loss occurred in 20.7% (6/29) and 25.0% (8/32) of the patients in the ESD and ITS groups, respectively. However, the differences were not statistically significant (*χ*^2^ = 2.452, *p* > 0.05; Figure 4B).

### Tinnitus improvement

**Figure 5:**
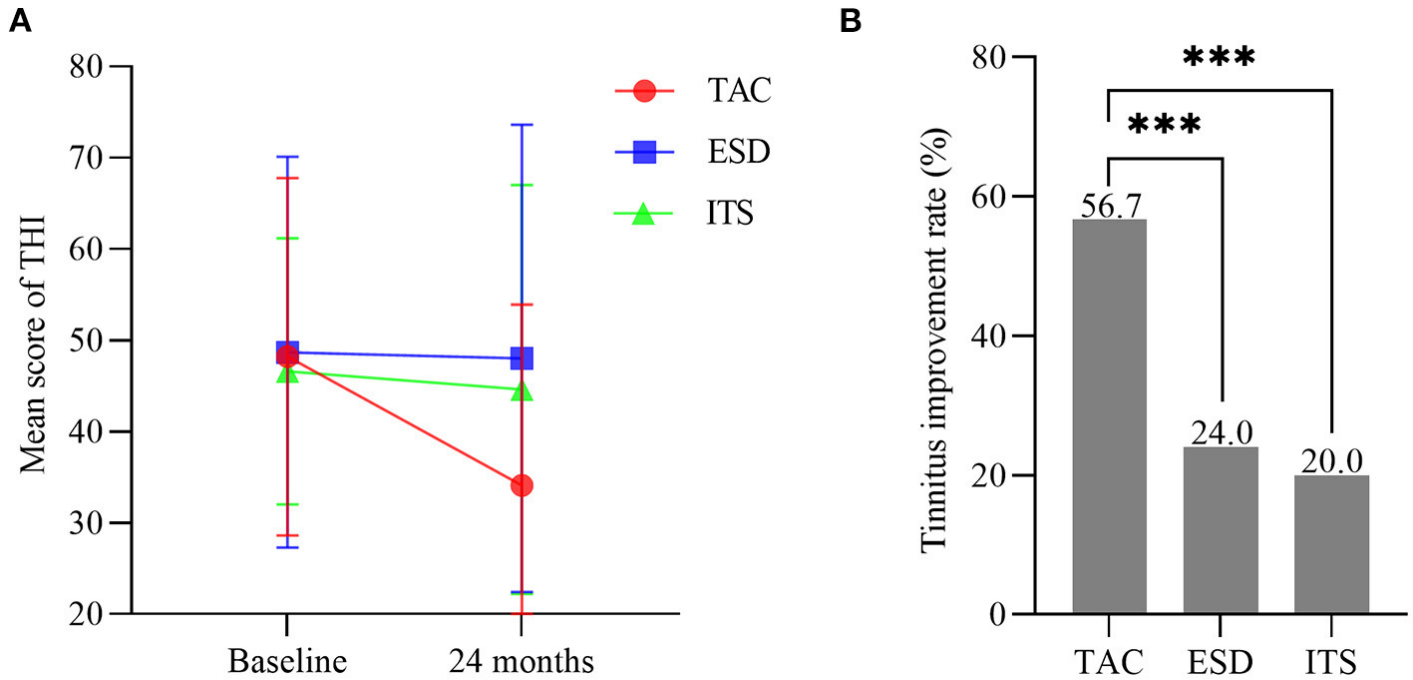
**(A)** Mean scores for tinnitus handicap inventory (bars are SDs). **(B)** The tinnitus improvement rate in each group (***p < 0.001). TAC, tympanic antrum catheterization; ESD, endolymphatic sac decompression; ITS, intratympanic steroid.

No significant differences were noted among the three groups for the THI score at baseline (*F* = 0.165, *p* > 0.05). With time, the mean score for THI in patients with TAC decreased from 48.2 ± 19.6 to 34.1 ± 19.8 (*t* = 3.927, *p* < 0.001; Figure 5A), with a significantly lower THI score at 24 months for the TAC group than for the ESD and ITS groups (*t* = 3.214, *p* < 0.01; *t* = 2.632, *p* < 0.05). The tinnitus improvement rate in patients with TAC was 56.7% (34/60). In the ESD and ITS groups, tinnitus improved in 24.0% (12/50) and 20.0% (10/50) of the patients, respectively. The tinnitus improvement rate in the TAC group was significantly higher than that in the ESD and ITS groups (*χ*^2^ = 11.962, *p* < 0.001; *χ*^2^ = 15.278, *p* < 0.001; Figure 5B).

### Aural fullness improvement

**Figure 6:**
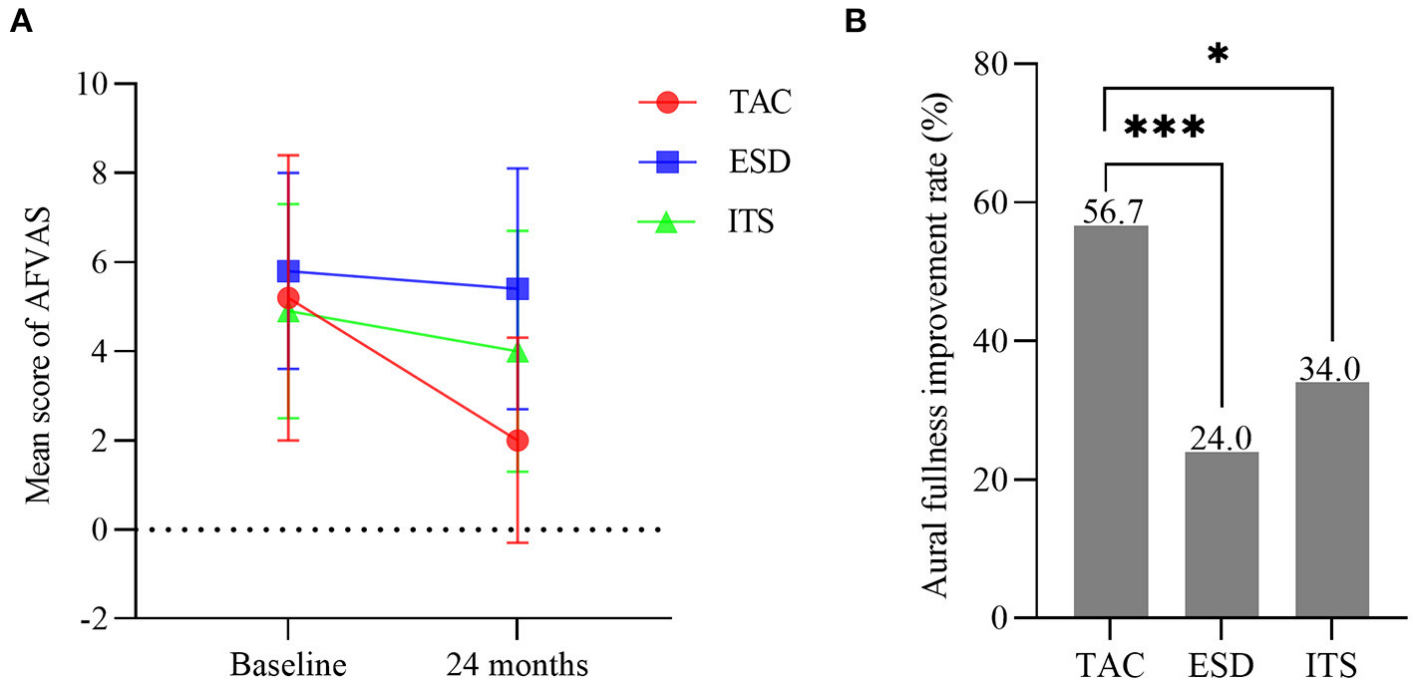
**(A)** Mean scores for aural fullness visual analog scale (bars are SDs). **(B)** The aural fullness improvement rate in each group (*p < 0.05, ***p < 0.001). TAC, tympanic antrum catheterization; ESD, endolymphatic sac decompression; ITS, intratympanic steroid.

One-way ANOVA showed no significant difference among groups for the AFVAS score at baseline (*F* = 1.299, *p* > 0.05; Figure 6A). With 2-year follow-up, the mean score for AFVAS in patients with TAC decreased from 5.2 ± 3.2 to 2.0 ± 2.3 (*t* = 6.446, *p* < 0.001), with a significantly lower AFVAS score at 24 months for the TAC group than for the ESD and ITS groups (*t* = 7.154, *p* < 0.001; *t* = 4.181, *p* < 0.001). The aural fullness improvement rate in patients with TAC was 56.7% (34/60). In the ESD and ITS groups, improvement in aural fullness occurred in 24.0% (12/50) and 34.0% (17/50) of the patients, respectively. The aural fullness improvement rate in the TAC group was significantly higher than that in the ESD and ITS groups (*χ*^2^ = 11.962, *p* < 0.001; *χ*^2^ = 5.635, *p* < 0.05; Figure 6B).

### Functional level improvement

**Figure 7:**
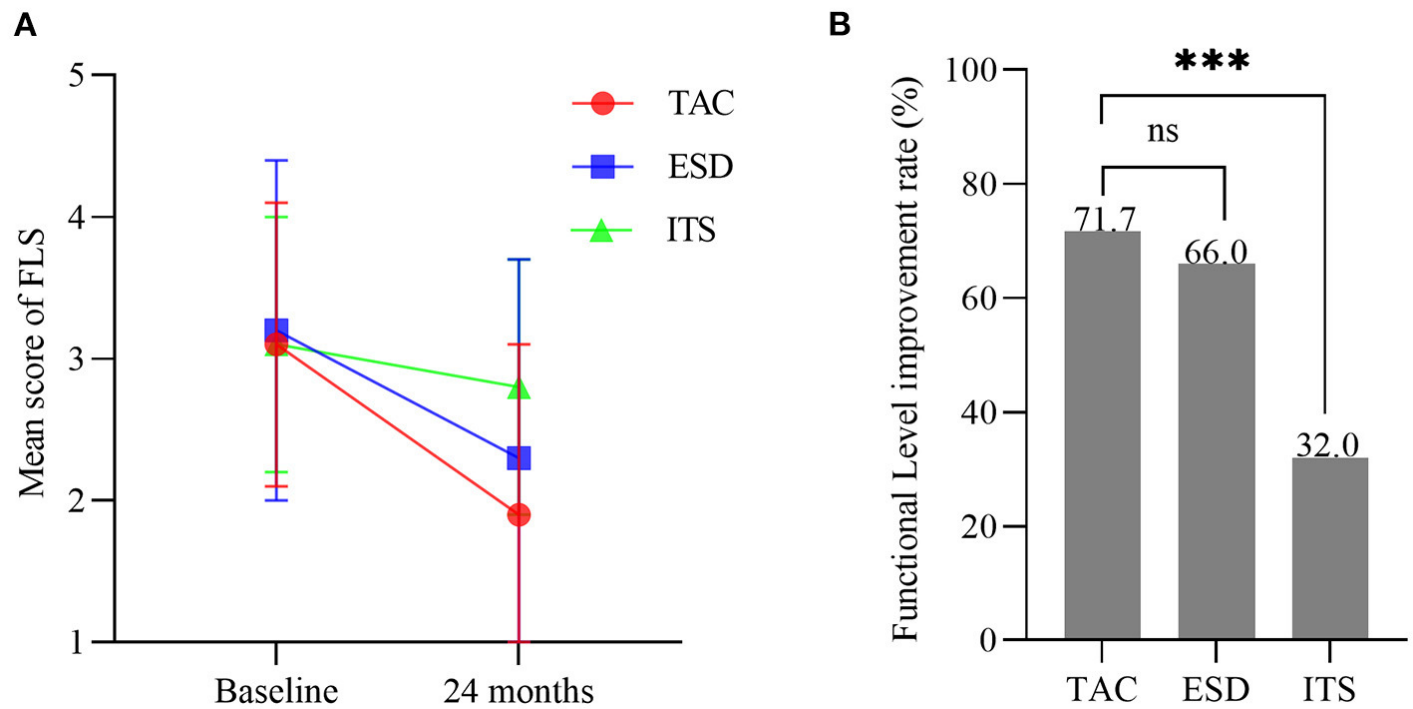
**(A)** Mean scores for functional level scale (bars are SDs). **(B)** The functional level improvement rate in each group (***p < 0.001). TAC, tympanic antrum catheterization; ESD, endolymphatic sac decompression; ITS, intratympanic steroid.

Baseline FLS score did not differ among the three groups (*F* = 0.384, *p* > 0.05). The mean score for FLS decreased over the follow-up period in all groups, with significant differences among the groups (*F* = 9.912, *p* < 0.001; Figure 7A). FLS score in patients with TAC decreased from 3.1 ± 1.0 to 1.9 ± 1.2 (*t* = 6.419, *p* < 0.001), which was significantly lower than that in the ITS group (*t* = 4.912, *p* < 0.001), but not statistically different compared to the ESD group (*t* = 1.644, *p* > 0.05). Moreover, the functional level improved in 71.7% (43/60) of patients in the TAC group and 66.0% (33/50) and 32.0% (16/50) of patients in the ESD and ITS groups, respectively. The functional level improvement rate of TAC was much higher than that of ITS (*χ*^2^ = 17.256, *p* < 0.001; Figure 7B), but no significant difference was found between TAC and ESD (*χ*^2^ = 0.410, *p* > 0.05).

### Adverse reactions and complications

There was no facial nerve injury, cerebrospinal fluid leakage, encephalic hematoma or infection in patients following TAC or ESD treatment. One case of tympanic membrane perforation occurred in ITS group, with an incidence of 2%.

## Discussion

Although there is still no cure for MD, over four-fifths of patients are alleviated from this disorder through lifestyle modification or medications. Surgical interventions such as ESD, semicircular canal plugging and labyrinthectomy are taken into account for patients with refractory MD who fail to control vertigo conservatively. However, these surgeries are performed under general anesthesia, with high physical condition requirements and poor patient acceptance, and are accompanied by a variety of post-operative complications and the risk of hearing loss.

In addition, there are various topical medication methods, such as tympanic injection, retroauricular injection, and sustained-release pumps. Retroauricular injection has widely used in the clinical management of sudden deafness (22). The dose of administration *via* intratympanic injection is limited due to the volume of the tympanic chamber. Besides, repeated tympanic membrane punctures increase patients' pain and fear, as well as the risk of tympanic membrane perforation, otitis media, and hearing loss (23). Animal studies have demonstrated that the osmotic pump provides a continuous long-term drug delivery to the cochlea (24), but it has not yet been applied on a large scale because of the lack of clinical data support and the high cost.

Therefore, drug administration *via* the tympanic antrum catheter was conceived to make up for aforementioned shortcomings and to allow for topical administration of multiple drugs. First, TAC has the advantage of topical medication, which avoids the blood-labyrinth barrier to maintain a higher drug concentration in the inner ear and avoids the side effects of systemic application. Second, TAC was performed under local anesthesia with less risk. The average operation time of TAC was significantly shorter than that of ESD (TAC, 34.7 ± 6.7 min; ESD, 75.3 ± 19.9 min; *t* = 13.808, *p* < 0.001). Third, TAC had essentially no damage to hearing level, except from a 2.4 ± 10.7 dB transient threshold shift of the affected ear, which was significantly lower than the 10.3 ± 11.2 dB of the ESD group (*t* = 3.799, *p* < 0.001). Notably, the cost of surgery in patients with TAC was significantly lower than that of the ESD group (TAC, 2,161.3 ± 201.4 yuan; ESD, 5,355.2 ± 302.7 yuan; *t* = 63.627, *p* < 0.001), which was more economical and acceptable for patients. Compared with ITS, single placement of TAC greatly reduced the painful experience of repeated punctures. Additionally, TAC is more widely indicated, regardless of age, stage, and unilateral/bilateral MD, for patients in whom conservative treatment is ineffective or who cannot undergo general anesthetic surgery. In particular, it can be re-catheterized for recurrence. The results of this study indicate that 76.7% of 60 intractable MD patients with TAC had effective vertigo control at the 2 years follow-up. Comparable vertigo control rate was observed in the ESD group, which is in line with our previous results (25). The rate of vertigo control with ITS was 58% in our study, which is within the range reported in previous studies (26). Moreover, TAC has significant advantages in improving tinnitus, aural fullness, and patients' functional levels, suggesting that TAC is an effective treatment of intractable MD. The mechanisms of action that we speculate on are as follows.

First, studies have shown that immunity and allergy are closely related to the pathogenesis of MD. There are higher rates of allergy history and immunologic components in patients with MD (27). Published studies have demonstrated the benefits of immunotherapy or dietary restrictions for the improvement of MD symptoms (28). Glucocorticoids are widely used in the clinical treatment of immune-related diseases owing to their anti-inflammatory and immuno-suppressive effects. Froehlich et al. (29) found widespread presence of glucocorticoid receptors in the inner ear, particularly in hair cells, spiral ligaments, and spiral ganglia. Previous studies have shown that local glucocorticoid administration is effective in the treatment of immune-mediated inner ear diseases such as sudden deafness and MD (30, 31). Glucocorticoids specifically bind to receptors to modulate the expression of inflammatory factors, thereby exerting anti-inflammatory and immunosuppressive effects (32). Glucocorticoids maintain endolymphatic fluid homeostasis by regulating aquaporin and Na^+^/K^+^-ATPase (33), thus reducing endolymphatic hydrops and vertigo attacks. The multiple effects of glucocorticoids, such as inhibition of hair cells senescence and apoptosis, neuroprotection, and antioxidant activity (34), might relieve the symptoms of tinnitus and aural fullness and protect against hearing loss.

There is evidence that MD is associated with middle ear pressure, which influences endolymphatic hydrops (35). Albu et al. (36) reported that, tenotomy of the middle ear muscles tendons blocked the active compression of stapedial footplate on oval window, thereby reducing inner ear hydrops associated with MD. Meniett device, a non-destructive, effective and portable therapy for intractable MD, generates low-pressure alternating pulses and activates the Salt–Rask-Anderson valve in the inner, thereby facilitating the resorbation of excess endolymph (37). Recently, Shojaku et al. (38) reported transtympanic membrane massage device, a new non-invasive treatment for MD, transmitted pressure change to the inner ear *via* the middle ear cavity. Therefore, we suggest that changes in pressure in the middle ear or changes in ossicular chain tension can affect inner ear pressure through the vestibular window or round window. The endolymphatic hydrops could be decompressed by reduction of ossicular chain tension, or could be relieved by the massaging effect of low-pressure pulse, thereby raising the threshold for vertigo attack and attenuating the damage to cochlear hair cells. Similarly, TAC may play a role in the low-pressure pulse effect through tube insertion and repeated drug infusion.

Moreover, Thomsen et al. (39) presented a prospective double-blind study of endolymphatic sac mastoid shunt and mastoidectomy in treatment of MD and demonstrated minor differences. The results of the study call into question the effectiveness of endolymphatic sac surgery, but from another point of view, it also confirms the effectiveness of mastoidectomy in the treatment of MD. Sajjadi and Paparella (40) indicated that mastoidectomy can indirectly decompress the endolymphatic sac through Trautmann's triangle. We speculate that the vibration of the inner ear triggered by the electric drill during mastoid surgery might also add to the treating effect of mastoidectomy by some means. Therefore, TAC may have a positive effect on MD symptom improvement *via* simple mastoidectomy, although the underlying mechanisms still need to be further explored.

As a preliminary study, a major limitation was the retrospective design of the study and the non-random nature of patient recruitment. And due to certain personal reasons and the COVID-19 epidemic, a limited number of patients came back to the hospital for retests of hearing and vestibular function. However, we obtained enough follow-up data in the form of interviews and questionnaires *via* telephone and E-mail.

## Conclusion

In conclusion, TAC has definite long-term effects in the treatment of patients with intractable MD. It had a valid effect on vertigo control and hearing preservation and showed significant advantages in improving tinnitus, aural fullness, and the overall functional level. Further studies are required to optimize the treatment in term of the type, dosage, and course of drug administration. We believe that TAC could be a promising novel modality for treating intractable MD.

## Author contributions

DZ, HW, and ZF designed the study. DZ, ZF, and YLy performed surgeries. YLy, JG, XL, HJ, YLi, and JW collected clinical data. JG, YLy, and XL performed data analysis and interpretation. YLy, JG, and DZ completed the manuscript. All authors contributed to the study and approved it for submission.

## Funding

This study was funded by the National Natural Science Foundation of China (No. 82171150), the Major Fundamental Research Program of the Natural Science Foundation of Shandong Province, China (No. ZR2021ZD40), Shandong Provincial Natural Science Foundation (No. ZR2020MH179), and Taishan Scholars Program of Shandong Province (No. ts20130913).

## Conflict of interest

The authors declare that the research was conducted in the absence of any commercial or financial relationships that could be construed as a potential conflict of interest.

## Publisher's note

All claims expressed in this article are solely those of the authors and do not necessarily represent those of their affiliated organizations, or those of the publisher, the editors and the reviewers. Any product that may be evaluated in this article, or claim that may be made by its manufacturer, is not guaranteed or endorsed by the publisher.
